# Experimental evidence for pigs as a potential long-term reservoir of *Trypanosoma brucei gambiense*

**DOI:** 10.1051/parasite/2026061

**Published:** 2026-10-28

**Authors:** Kouka Zoéwentallé Thibaut Ouédraogo, Jacques Kabore, Kadidiata Ilboudo, Alain Boulangé, Sophie Thevenon, Robert Eustache Hounyeme, Hassane Sakandé, Mohamed Bamba, Geoffrey Gimonneau, Brice Rotureau, Jean-Mathieu Bart, Adrien Gaston Marie Belem, Bruno Bucheton, Marc Desquesnes, David Berthier-Teyssedre, Veerle Lejon, Vincent Jamonneau

**Affiliations:** 1 Unité de Recherche sur les Maladies à Vecteurs et Biodiversité, Centre International de Recherche-Développement sur l’Élevage en Zone Subhumide 01 BP 454 Bobo-Dioulasso 01 Burkina Faso; 2 Unité de Formation et de Recherche Sciences et Techniques, Université Nazi Boni 01 BP 1091 Bobo-Dioulasso Burkina-Faso; 3 Unité de Recherche « Trypanosomoses », Institut Pierre Richet 01 BP 1500 Bouaké Côte d’Ivoire; 4 INTERTRYP, Univ Montpellier, Cirad, IRD 34980 Montferrier-sur-Lez France; 5 CIRAD, UMR INTERTRYP 34398 Montpellier France; 6 Parasitology Unit, Institut Pasteur of Guinea, Route de Donka BP 4416 Conakry Guinea; 7 Institut du Développement Rural, Université Nazi Boni 01 BP 1091 Bobo-Dioulasso Burkina-Faso; 8 Université de Toulouse, ENVT, INRAE, IHAP, 23 Chemin des Capelles 31300 Toulouse France

**Keywords:** Human African trypanosomiasis, Sleeping sickness, Diagnosis, Pig, Experimental infection

## Abstract

Pigs are suspected to act as a reservoir for *Trypanosoma brucei gambiense* and may therefore contribute to the re-emergence of human African trypanosomiasis (HAT), despite ongoing efforts to eliminate transmission. To clarify the role of this species in HAT epidemiology and to better understand the dynamics of *T. b. gambiense* infection in blood, we experimentally infected pigs with this parasite. In this study, we compared nine infected pigs with eight control pigs over a period of more than ten months using parasitological, molecular, and serological methods. The results showed that each diagnostic method has limitations, in terms of sensitivity to detect the presence of *T. b. gambiense*, and specificity to differentiate infection caused by *T. b. gambiense* from infection caused by *T. b. brucei,* responsible for animal African trypanosomosis. Nevertheless, the overall analysis showed that pigs can remain infected with *T. b. gambiense* for at least ten months. Hence, pigs should be considered a potential long-term reservoir of *T. b. gambiense* that could compromise the sustainability of the current elimination process. As a result, there is a need to pursue investigations on this topic, considering possible co-infections with *T. b. brucei*. In the meantime, adapted measures should be envisaged to limit contact between pigs and tsetse flies and/or to clear potential pig infections with *T. b. gambiense* by curative treatment.

## Abstract (fr)

Les porcs sont soupçonnés de servir de réservoir à *Trypanosoma brucei gambiense*, à partir duquel la trypanosomose humaine africaine (THA) pourrait réémerger alors même que la maladie fait actuellement l'objet d'un objectif d'élimination de la transmission. Afin d'élucider le rôle de cet animal dans l'épidémiologie de la THA et de mieux comprendre la dynamique de l'infection sanguine, nous avons infecté expérimentalement des porcs avec *T. b. gambiense*. L'étude a comparé neuf porcs infectés et huit porcs témoins sur une période de plus de dix mois, en utilisant des méthodes parasitologiques, moléculaires et sérologiques. Les résultats ont révélé que chaque méthode diagnostique présente des limites, tant en termes de sensibilité pour détecter la présence de *T. b. gambiense* que de spécificité pour distinguer une infection par *T. b. gambiense* d'une infection par *T. b. brucei*, responsable de la trypanosomose animale africaine. Néanmoins, l'analyse globale a montré que les porcs peuvent rester infectés par *T. b. gambiense* pendant au moins dix mois. Par conséquent, les porcs doivent être considérés comme un réservoir potentiel à long terme de *T. b. gambiense*, susceptible de compromettre la pérennité du processus d'élimination en cours. Il est donc nécessaire de poursuivre les recherches sur ce sujet, en prenant en compte les éventuelles co-infections par *T. b. brucei*. Dans l'intervalle, des mesures adaptées devraient être envisagées pour limiter les contacts entre les porcs et les mouches tsé-tsé et/ou pour éliminer les infections porcines potentielles à *T. b. gambiense* par un traitement curatif.

## Introduction

Human African trypanosomiasis (HAT), caused by *Trypanosoma brucei gambiense* (*T. b. gambiense*), used to be a major public health problem in intertropical Africa [28]. Large-scale medical surveillance and treatment campaigns had brought the disease close to elimination by the 1960s. Control subsequently lapsed amid an early sense of victory, political instability, and reduced funding, allowing a resurgence that peaked in the 1990s. Renewed international control efforts then gradually reduced the disease burden from the early 2000s onwards [17, 28]. Thanks to sustained control efforts including medical surveys, treatment, and vector control, the prevalence of this disease is currently at its lowest reported level. Gambiense HAT is now targeted for elimination of transmission by 2030 [17].

However, the existence of a potential animal reservoir and its epidemiological role could compromise efforts to eliminate the disease [4]. The role of domestic pigs in the maintenance and/or re-emergence of the disease is currently actively debated. Recent studies, in endemic and historical HAT foci in Côte d’Ivoire and Cameroon, have shown that pigs are multi-infected by trypanosomes including *T. brucei* [15, 29, 49]. Possible explanations include the following: (i) pigs often range freely in biotopes favourable to tsetse fly on the outskirts of anthropised areas [39, 49], (ii) pigs are the preferred host of riverine tsetse flies, especially *Glossina palpalis palpalis*, the main vector of *T. brucei* subspecies [44], and (iii) pigs are susceptible to, and tolerant of, trypanosome infections [25, 47]. Moreover, most of these studies also confirmed the challenge of distinguishing the human pathogen *T. b. gambiense* from *T. b. brucei*, which causes animal African trypanosomosis (AAT), highlighting the lack of tools to prove or exclude the presence of *T. b. gambiense* in these animals.

Parasitological methods have limited sensitivity and are unable to differentiate *T. brucei* subspecies morphologically. Sensitive molecular methods, such as TBR PCR [36] or M18S-II PCR [11] are specific of the subgenus *Trypanozoon* and do not allow differentiation between *T. b. gambiense* and *T. b. brucei*. PCRs using the AnTat A/B and LiTat A/B primers [3] are also not specific for *T. b. gambiense* [19]. The PCR methods targeting the single copy gene coding for the *Trypanosoma gambiense-specific glycoprotein* (*TgsGP*), are considered specific for *T. b. gambiense* [42]. However, they lack sensitivity and are prone to false positivity through the amplification of TgsGP-like sequences or contamination especially when using a nested PCR format to increase sensitivity [9, 19, 49].

Moreover, the diagnostic accuracy of existing *T. b. gambiense* serological field tests in animals remains poorly elucidated. Recent field or experimental studies conducted on pigs have shown that the Card Agglutination Test for Trypanosomiasis*/T*. *b*. *gambiense* (CATT) [30] and rapid diagnostic tests (RDT) such as the HAT Sero-K-SeT [5] and the SD Bioline HAT [2] are not specific to *T. b. gambiense* [20, 33, 49, 53]. The immune trypanolysis test (TL) [52] is incorporated into the standard HAT surveillance diagnostic algorithm [10, 38]. However, its reliability for use with animals has been questioned with (i) high positivity rates in pigs that are inconsistent with the low HAT prevalence in Côte d’Ivoire [39, 49], (ii) positive TL results observed in cattle in a region of Uganda where *T. b. rhodesiense* HAT and AAT endemic [33], and (iii) positive TL results observed in pigs experimentally infected with *T. b. brucei* [21]. The indirect enzyme-linked immunosorbent assay (indirect ELISA) could also be a promising immunological tool for the detection of *T. b. gambiense* [38], but limited data are available in animals.

To clarify the role of domestic pigs in HAT epidemiology, we conducted experimental pig infections with *T. b. brucei* and *T. b. gambiense*. The aim was to improve our understanding of the dynamics of infection of these two subspecies in pigs using the available diagnostic tools to directly or indirectly detect the presence of *T. b. gambiense*. Infection with *T. b. brucei* was conducted in 2020 [20, 21]. Results indicate that, depending on the context, the objectives, and the available resources, most HAT serological methods are suitable for *T. b. brucei* AAT surveillance. However, they also confirmed that serological tests used for HAT are not specific to *T. b. gambiense*, limiting their relevance for studies in animals. The results of the experimental *T. b. gambiense* infections in pigs conducted in 2022 are reported in the present article.

## Materials and methods

### Ethics statement and biosafety

Approval and authorisation for implementation (protocol number 070_2020/ADM/DG/nka) was received from the CIRDES Ethics and Biosafety Committee. To mitigate biosafety risks, the pigs were housed in a secure and disinfected barn protected by insecticide-impregnated “ZeroFly™” netting from Vestergaard^®^. Insecticide-impregnated tsetse traps were installed around the barn, and an airlock was maintained at the main entrance to prevent insect intrusion. A veterinary technician performed all sampling and checked the general conditions of the animals at each sampling visit (see below). Protocols were in place for immediate euthanasia of any animal showing signs of distress or abnormal trends in critical measurements (weight loss, PCV significant decrease). As there is not effective or recommended treatment for *T. b. gambiense* in pigs, all animals were euthanised at the end of the experimental period.

### Experimental infection

Pigs were infected with the B4F303 strain, isolated in 2004 from a HAT case in the Bonon focus in Côte d’Ivoire and genetically characterised as *T. b. gambiense* [26]. This strain was chosen due to its moderate virulence and pathogenicity previously characterised in a mouse model [24]. The strain, stored in liquid nitrogen, was reactivated by thawing of the stabilate at 37 °C. After checking under a microscope that the trypanosomes were still alive, 100 μL of the sample diluted 1:10 in sterile 1% phosphate-buffered saline with glucose (PSG) were intraperitoneally inoculated into four Naval Medical Research Institute (NMRI) mice for parasite amplification. The mice were immunosuppressed 24 hours before infection by a subcutaneous injection of 0.2 mL of cyclophosphamide (Endoxan™, 300 mg/kg).

Parasitaemia was monitored daily by tail-tip blood sampling using the matching method [18]. When the parasitaemia reached a minimum score of 7.8 (16 trypanosomes/field ≈ 64 × 10^6^ trypanosomes/mL), the mice were anaesthetised with an intraperitoneal injection of ketamine (50 mg/kg of live weight) and bled by cardiac puncture. The collected blood was then pooled and diluted in sterile PSG to achieve a concentration of approximately 10^5^ trypanosomes/mL.

Seventeen male, uncastrated mixed-breed pigs (Large White, Hampshire, and Duroc crosses), aged 3–6 months and weighing 10–28 kg, were acquired from a farm on the outskirts of Bobo-Dioulasso, Burkina Faso. After their arrival at the CIRDES barn under mosquito nets (21/12/2021), all animals received blanket treatments with an intestinal deworming treatment (levamisole hydrochloride 8 mg/kg in drinking water, 21/12 and 24/12/2021), an antibiotic (oxytetracycline 0.1 mL/kg body weight, injected intramuscularly, 24/12 and 27/12/2021), followed by a trypanocide (diminazene aceturate 7 mg/kg body weight, injected intramuscularly, 04/01/2022).

Six weeks after the diminazene aceturate treatment, nine pigs were randomly selected and inoculated with two 500 μL intradermal injections of a suspension containing 10^5^ trypanosomes/mL, in the left and right flank. The remaining eight uninfected pigs were used as the control group. As for the *T. b. brucei* infection, 6 months of follow-up was planned. However, the results prompted us to extend the monitoring period to 10 months (305 days) for certain animals with the ethics committee’s agreement.

### Sampling

A volume of 500 μL of the 10^5^ trypanosomes/mL suspension of the strain amplified in mouse (SSAM) and diluted in PSG (SSAM-PSG sample) was stored at −20 °C for subsequent diagnostic PCRs for infection strain control.

For pig sampling, we considered the day of infection, D0, as the basis. The samples collected X days before infection were labelled −X DPI. Those collected X days post-infection were labelled X DPI.

Blood samples were collected in 5 mL heparinised Vacutainer^®^ tubes from the central vascular system (anterior vena cava) at −59 DPI (upon arrival at CIRDES), −17, −10, −3 DPI, and D0 (day of infection), then twice a week until 249 DPI, and weekly until the end of follow-up (305 DPI). From the blood samples collected at −59, −17, −10, and −3 DPI, and weekly from 4 to 305 DPI, 500 μL buffy coat (BC) and 1 mL plasma were collected after centrifugation at 15,000 rpm for 15 min. These samples were stored at −20 °C for subsequent serological and molecular analyses.

### Clinical monitoring

A veterinary technician systematically recorded the clinical manifestations observed during follow-up, checking the general condition of the animals at each sampling visit after infection. The packed cell volume (PCV) of each animal was measured using heparinised capillary tubes collected from the heparinised Vacutainer^®^ tubes, after centrifugation at 10,000 rpm for 5 min. The weight of the pigs was recorded weekly from −56 DPI onwards.

### Diagnostic tests

For parasitological examination, all capillary tubes collected for PCV were examined by the buffy coat technique (BCT) [37]. In case of a BCT-positive result, parasitaemia was first estimated following a matching method [18] and then determined using the method described by Paris *et al.* (1982) [40].

For molecular diagnosis, DNA from the SSAM sample and from all Buffy Coat (BC) samples (except those collected at −59 DPI) was extracted using a DNeasy Blood & Tissue Kit (QIAGEN, Valencia, CA, USA). DNA from BC samples (including those collected at −59 DPI) were also processed using the Chelex 100 DNA preparation method [54]. SSAM DNA was tested with TBR (primers TBR1/2) [36] and TgsGP (primers TgsGP1/2) single round PCR [42]. All DNA from BC samples was tested with the TBR, TgsGP single round, TgsGP nested [35], M18S-II [11], and the AnTat A/B and LiTat A/B [3] recently readapted [19] PCR. DNA from the −59 DPI BC samples was also tested using PCR assays targeting *T. vivax* (primers TVW1/2) [32], *T. congolense* forest type (primers TCF1/2), and *T. congolense* savannah type (primers TCS1/2) [31].

For serological diagnosis, plasma was tested using the HAT Sero-*K*-Set RDT (Coris BioConcept, Gambloux, Belgium) [5], CATT (Institute of Tropical Medicine Belgium) [30], indirect ELISA *T. b. brucei* and TL using the VAT LiTat 1.3, LiTat 1.5, and LiTat 1.6 [22, 52] as previously described [20, 21]. For indirect ELISA, we decided to use the ELISA/*T. b. brucei* already standardised for the analysis of pig plasmas [20]. The ELISA positivity threshold was set at an optical density (OD) of 0.167, corresponding to the mean OD values plus five standard deviations from all the control pig samples and all the samples collected before infection from the infected group. Indirect ELISA tests with lysates of *T. vivax* and *T. congolense* [12, 16] were also performed on samples collected at −59 DPI.

### Statistical analysis

Data were recorded in Microsoft Excel 2019 and analysed using R software (version 4.5.2). Graphs were generated using the *ggplot* function from the *ggplot2* package [55]. Kaplan–Meier survival analysis [14] and the log-rank test were used to compare survival curves between the two pig groups using the survival package. The effects of infection on weight and PCV were analysed using mixed-effects models (pigs as random effects), with comparisons performed using lsmeans. The final model, fitted in R using the nlme package, was selected based on Akaike’s information criterion (AIC) and Bayesian information criterion (BIC).

The specificity of serological and molecular tests, defined as their ability to produce a negative result in a sample from an uninfected animal, was assessed on samples from pigs of the control group and from the infected group tested prior to infection including D0. The sensitivity of the tests, defined as their ability to produce a positive result in a sample from an infected pig, was assessed on two time periods: from 4 to 28 DPI to evaluate the potential for early detection of infection and from 32 DPI (at least one BCT-positive result observed in each pig confirming an effective infection) to 147 DPI (at least four surviving pigs). The specificity and sensitivity and their confidence interval (CI) were assessed with the binomial test [46]. The sensitivity of the PCR using the two extraction methods was compared using the McNemar test. The files containing the raw data, supplementary figures, and the R script can be found in the Supplementary Materials.

## Results

### Clinical outcome and mortality

Clinical signs and symptoms observed in individual pigs at each visit are described in Supplementary Data 1. All pigs developed respiratory syndrome with symptoms as dyspnoea, cough, and nasal discharge (rhinorrhoea). Multiple inflammatory manifestations were observed, including dermatitis and adenitis. Most pigs also showed marked difficulty moving and remained recumbent for prolonged periods. Overall, pigs in the control and infected groups showed similar signs and symptoms. The clinical signs observed led to suspicion of several infectious diseases, including pasteurellosis, African swine fever, salmonellosis, babesiosis, swine coccidiosis, ascariasis, and colibacillosis. These diseases were investigated by the appropriate methods and only colibacillus cultures and tests for *Coccidia* and *Ascaris* were positive. Benzal (albendazole), PeniStrep (penicillin G procaine + dihydrostreptomycin) and Amprolium (amprolium Hydrochloride) were used to treat gastrointestinal infections, salmonellosis, and coccidiosis, respectively. Theracalcium (calcium gluconate + calcium glucoheptonate) was administered to prevent hypocalcaemia. These treatments were administered between 119 and 133 DPI. In the days following the treatment, *Ascaris* nematodes were observed in the faeces confirming this infection. The various symptoms then almost resolved until the end of the experiment. Diet was most likely the cause of these confirmed or suspected infections in the pigs in both groups.

More than half of the pigs had to be euthanised due to deteriorating health before the initial 6-month (180 DPI) study duration was reached. The experiment continued for up to 10 months (305 DPI) for surviving animals, as long as their health status allowed. Mortality was recorded during the follow-up period (Supplementary Data 2). Of the 17 pigs, only two could be followed up until 305 DPI before being euthanised. The survival curves of pigs regarding the two groups (infected/control) are presented in Figure 1. The mortality rate was similar between the two groups (*p*-value = 0.9).

**Figure 1:**
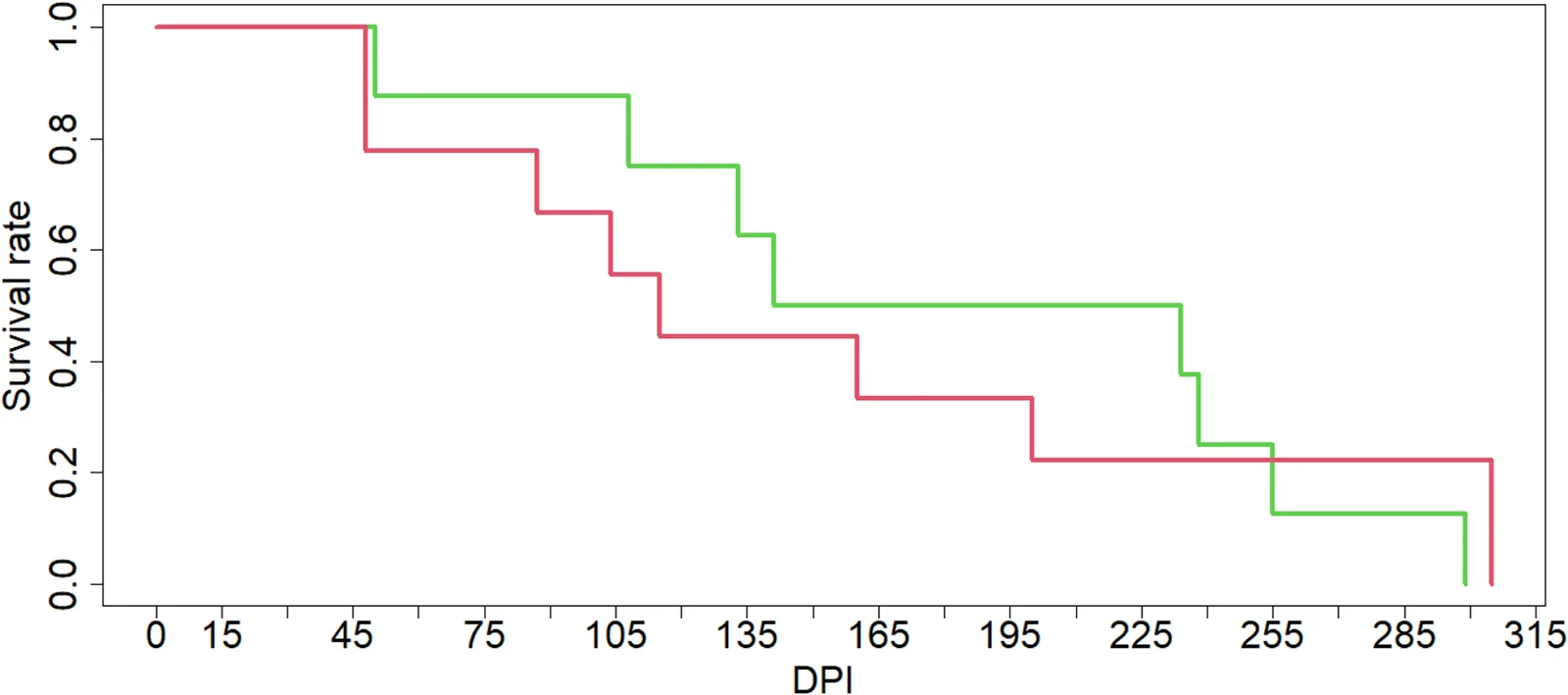
Survival curve of test pigs. Red line: infected group, green line: control group; DPI: days post-infection.

The packed cell volume (PCV, or haematocrit) values are presented in Supplementary Data 3. The average PCV of the pigs did not vary significantly during the follow-up period (*p-*value = 0.3). No significant difference was observed between the two groups during the post-infection period (*p*-value = 0.2) (Fig. 2). Low PCV values were occasionally observed in several pigs in both groups.

**Figure 2:**
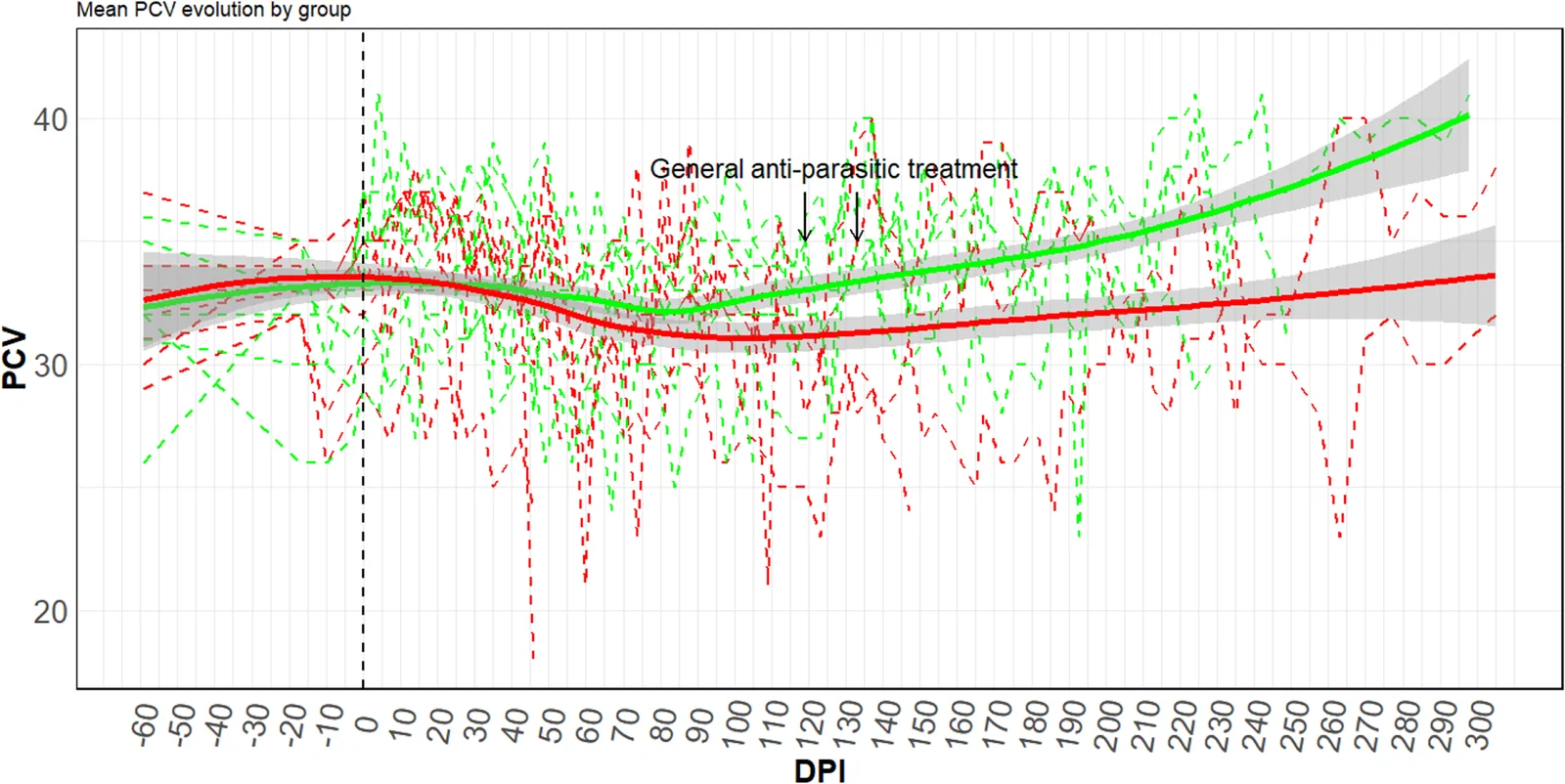
Changes in average packed cell volume of pigs in both groups during infection. PCV: packed cell volume; Red line: infected group, green line: control group; solid line: average PCV trends estimated using smoothing, with their 95% confidence intervals, dashed line: individual PCV trends for pigs; DPI: days post-infection, Treatment indicates those administered between 119 and 133 DPI to treat gastrointestinal infections, salmonellosis, and coccidiosis.

Weight data for individual pigs are presented in Supplementary Data 4. The average weight gain in both groups followed an upward trend during the experiment. No significant differences were observed between the two groups during the post-infection period (*p*-value = 0.8) (Fig. 3), the graphical difference being due to pig mortality.

**Figure 3:**
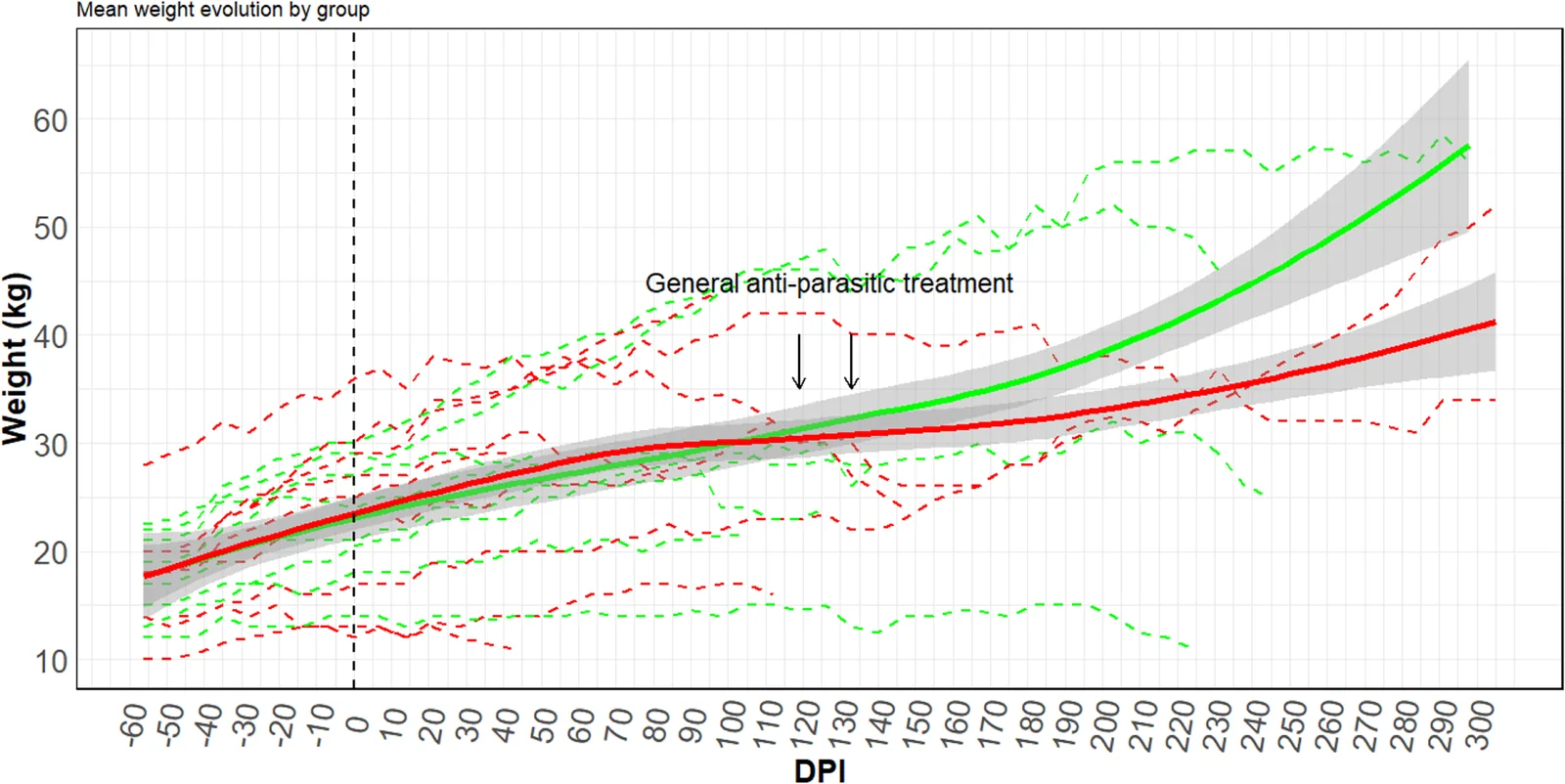
Evolution of weight of pigs in both groups during infection. Red line: infected group, green line: control group; solid line: average weight trends estimated using smoothing, with their 95% confidence intervals, dashed line: individual weight trends for pigs; DPI: days post-infection, Treatment indicates those administered between 119 and 133 DPI to treat gastrointestinal infections, salmonellosis, and coccidiosis.

### Parasitological monitoring

BCT results are given in Supplementary Data 5. All samples collected from the 17 pigs upon arrival at the CIRDES (−59 DPI) were negative. BCT was then performed on 837 samples during the experiment, including 68 pre-infection samples (17 pigs and four visits before infection, including D0) and 769 post-infection samples of which 367 were from the infected group and 402 from the control group. All samples collected before infection as well as those from the control group were BCT-negative. The BCT was positive for 92 samples from the infected group post-infection (92/367, 25% of the samples) (Fig. 4). All infected pigs showed positive BCT several times during infection (from 5 times for pig 10 to 22 times for pig 1). The pre-patent period ranged from 17 (pigs 1 and 24) to 32 DPI (pig 3), with an average time of 21 ± 4.4 DPI. Between 32 and 49 DPI, most of the infected pigs were BCT-positive (35 positive tests/52). From 53 DPI, the BCT positivity rate fluctuated and decreased among the seven surviving pigs. They all became negative before dying or being euthanised. Parasites remained detectable up to 182 DPI for pig 2. Of the two pigs followed up until 305 DPI, BCT became negative at 81 DPI (pig 24) and 137 DPI (pig 7).

**Figure 4:**
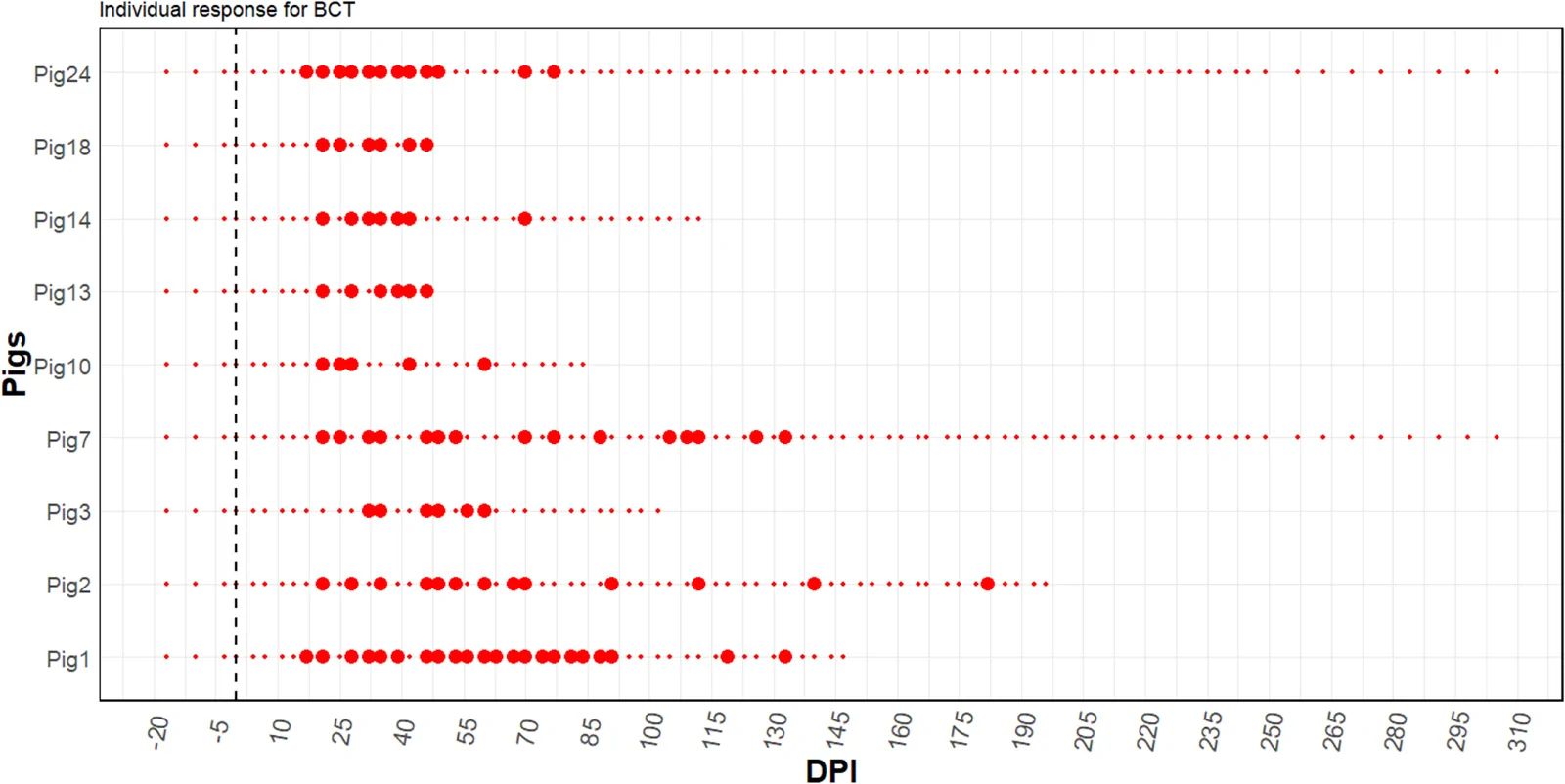
BCT results per infected pig. Large red dot: BCT-positive, small red dot: BCT-negative, empty: not available (dead animal); DPI: days post-infection.

Concerning the 92 BCT-positive results observed during the study, parasitaemia levels ranged from 2 × 10^3^ (59 observations) to 4 × 10^5^ trypanosomes/mL (one observation with pig 24 at DPI 21). Overall, parasitaemia levels were low, with an overall average of 5 × 10^3^ trypanosomes/mL. The average parasitaemia observed in BCT-positive pigs remained stable, ranging from 2 × 10^3^ to 5 × 10^4^ trypanosomes/mL throughout the infection (Fig. 5).

**Figure 5:**
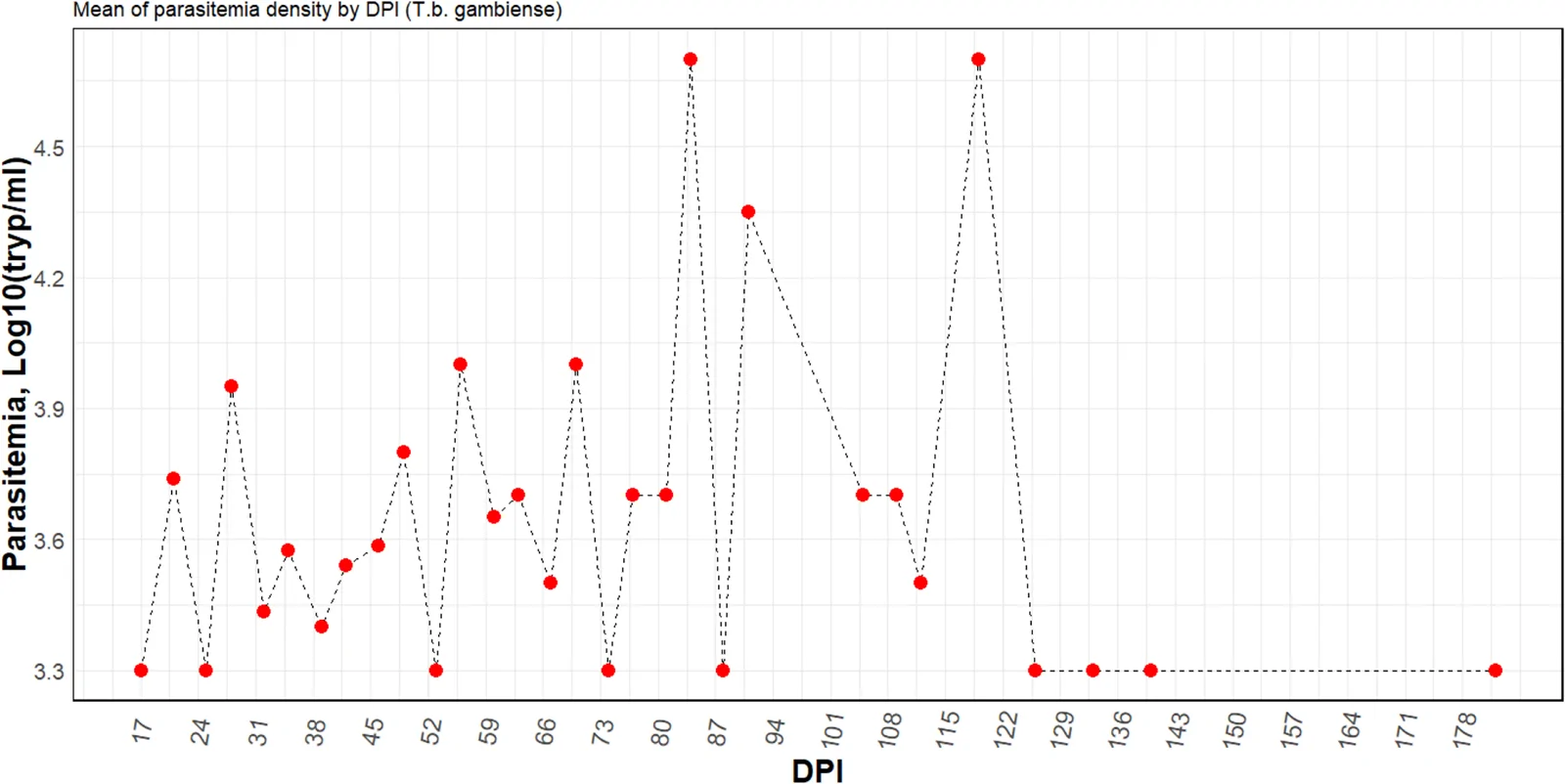
Changes in average parasitaemia in BCT-positive pigs. Red dots correspond to the average parasite density observed on the days post-infection (DPI) on which at least one pig was positive to BCT.

### Molecular monitoring

The SSAM-PSG DNA collected in previous experimental infection tested positive for both the TBR and TgsGP single round PCRs, confirming the status of the strain as a *T. b. gambiense* isolate. PCR results obtained from pig samples are provided in Supplementary Data 6. All PCRs performed on the samples collected from the 17 pigs upon arrival at the CIRDES (−59 DPI) were negative. PCRs were then performed on 452 samples during the study, including 51 pre-infection samples (17 pigs and three visits before infection) and 401 post-infection samples, of which 194 from the infected group and 207 from the control group. The results of each PCR according to the extraction method are illustrated in Supplementary Figures (S1 to S8). A summary of these results is presented in Table 1, focusing on (i) proportion of positive results on samples collected from the infected group post-infection, (ii) prepatent period indicating the period between the DPI of the first and the last positive pig, (iii) average DPI of the first positive result considering all positive pigs, and (iv) DPI of the last positive result.

**Table 1:** PCR results according to the extraction method for the infected pigs.

Extraction type	PCR	% Pos. post-inf (95% CI)	Prepatent period	Mean first pos. DPI	Last Pos. DPI
Chelex	TBR	32.9; 64/194 (26–40%)	11–32	22.22 (±8.14)	298
	single round TgsGP	0; 0/194 (0–10%)	–	–	–
DNeasy	TBR	74.7; 145/194 (68–81%)	4–25	14.4 (±5.9)	305
	single round TgsGP	2.6; 5/194 (1–6%)	60–151	105.5 (±64.5)	**213**
	Nested TgsGP	35.6; 69/194 (29–43%)	4–39	21.9 (±11.13)	**291**
	AntatA/B	8.2; 16/194 (5–13%)	17–88	41.9 (±24.4)	151
	LitatA/B	27.8; 54/194 (22–35%)	11–109	32.8 (±31.9)	242
	M18-S	39.7; 77/194 (33–47%)	4–53	22.4 (±16.3)	263

% Pos. post-inf: percentage of positive post-infection samples; First Pos. DPI: date of first positive test; Last Pos. DPI: date of last positive test.

Using the Chelex method, 100% (194/194) and 67.1% (130/194) of the post-infection samples tested negative in single round TgsGP and TBR PCR, respectively. Given the apparent low sensitivity of these two PCR reactions performed on DNA processed with Chelex, we decided not to test these samples with the other PCRs. To illustrate this choice, the sensitivity observed with the TBR PCR on samples extracted with the DNeasy Kit was significantly higher (*p* < 0.0001).

For DNA extracted using the DNeasy Kit, the TBR and M18S-II PCRs were positive for 2.7% (7/258) and 0.8% (2/258) of the samples collected before infection and from the control group, respectively. All these samples were negative with the other PCRs. TBR and the single round TgsGP PCRs were positive for 74.7% (145/194) and 2.6% (5/194) of post-infection samples, respectively (Table 1). The nested TgsGP PCR was positive for 35.6% (69/194) of these samples. The proportion of positive results obtained with AnTat A/B, LiTat A/B, and M18S-II PCR ranged from 8.2% (16/194) to 39.7% (77/194). For all pigs, there was at least one PCR-positive result during the infection with the TBR and nested TgsGP PCRs. AnTat A/B and M18S-II were negative for all samples collected from pig 18. This pig, as well as pig 3, were also negative with LiTat A/B PCR. The single round TgsGP PCR was only positive for 5 samples from two pigs (1 and 7).

The DPI of the first positive PCR result was highly variable and ranged from 4 (TBR, nested TgsGP, and M18S-II) to 60 DPI (single round TgsGP). This was also the case for the average DPI of the first positive PCR result ranging from 14.4 DPI for TBR to 105.5 DPI for single round TgsGP PCR. Most of the PCR-positive results were observed between 20 and 150 DPI. For the 2 pigs (7 and 24) surviving after 200 DPI, the TBR PCR detected parasite DNA until 305 DPI (16/16 and 2/16 PCR-positive time points, respectively) and nested TgsGP until 291 DPI (5/16 and 1/16 positive time points, respectively).

### Serological monitoring and immune response

The results of the RDT, CATT, ELISA, and TL tests are given in Supplementary Data 7. All serological tests performed on −59 DPI were negative, confirming that pigs were probably not infected by trypanosomes. A total of 452 samples were then tested during the experiment, including 51 pre-infection samples and 401 post-infection samples, including 194 from the infected group and 207 from the control group. Except for the CATT that was positive for one pig in the control group (pig 22 at days −17, −10 and −3), all tests were negative with the pre-infection and control group samples.

All infected pigs showed positive results with the RDT, CATT, and TL tests. The first antibodies were observed with RDT between 17 and 74 DPI (average time 42 ± 20.4 DPI), with CATT between 11 and 32 DPI (average time 22.3 ± 7.3 DPI) and with TL between 25 and 39 DPI (average time 27.6 ± 3.9 DPI) (Fig. 6). Considering the 3 VATs of TL, the first positive tests for TL LiTat 1.3 were observed between 25 and 39 DPI (average time 28.1 ± 5.1 DPI), for LiTat 1.5 between 25 and 32 DPI (average time 27.3 ± 3.5 DPI) and for LiTat 1.6 between 25 and 32 DPI (average time 27.3 ± 3.5 DPI). The detection of antibodies was almost simultaneous for the 3 VAT (Fig. 7). For ELISA testing, four of the nine infected pigs (10, 13, 14, and 18) showed negative results across the whole study (Fig. 6), all corresponding to pigs that died prematurely (between 48 and 115 DPI). Pig 3 that died at 104 DPI, and the four pigs that could be followed up beyond 115 DPI, showed ELISA-positive results. In these five pigs that tested positive in ELISA, antibodies were detected from between 25 and 39 DPI (average time 30.6 ± 5.9 DPI). All pigs for which antibodies were detected by the four serological tests remained positive until their death.

**Figure 6:**
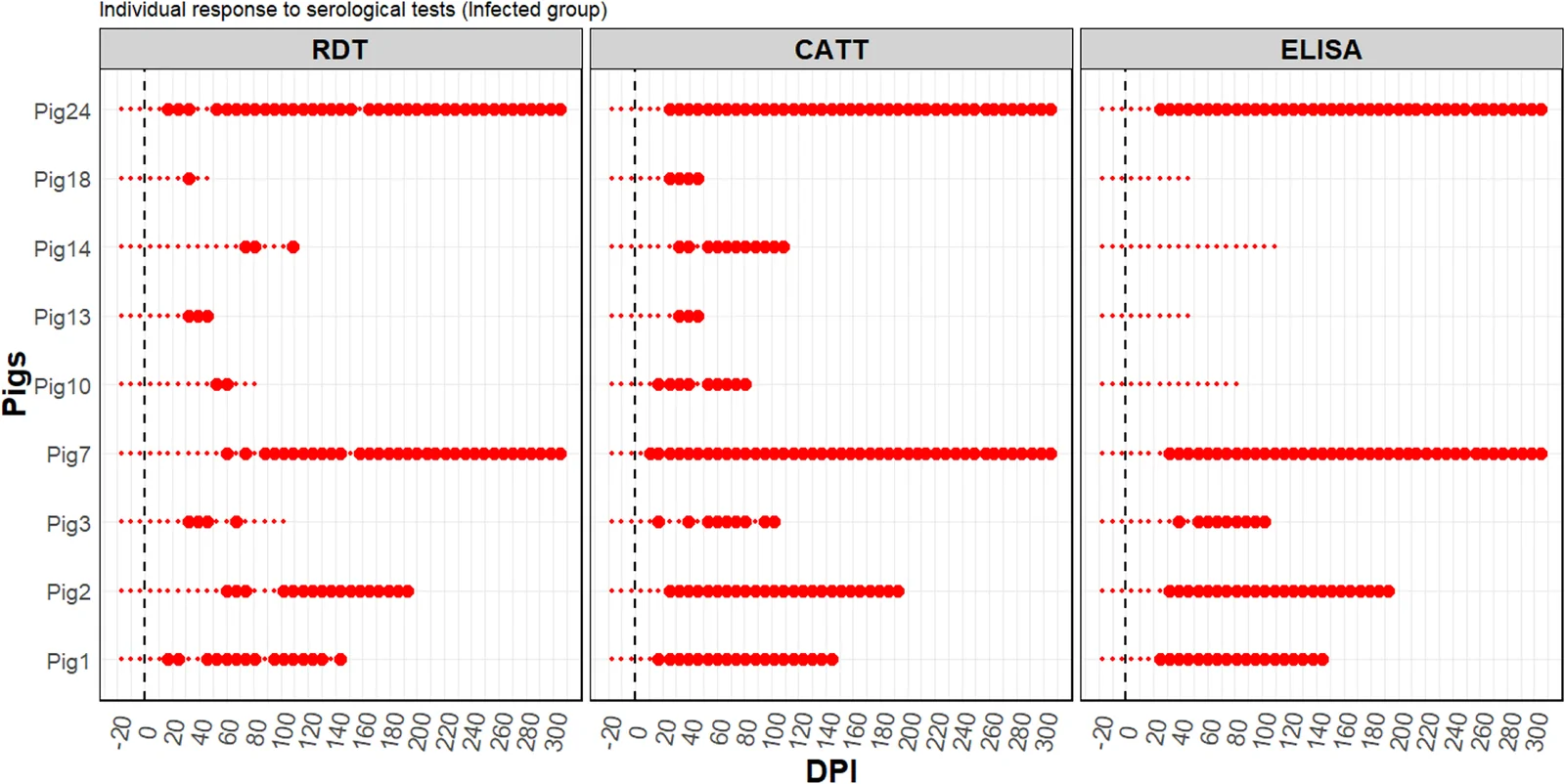
RDT, CATT, and ELISA results per infected pig. Large red dot: test positive, small red dot: test negative, empty: not available (dead animal); DPI: days post-infection.

**Figure 7:**
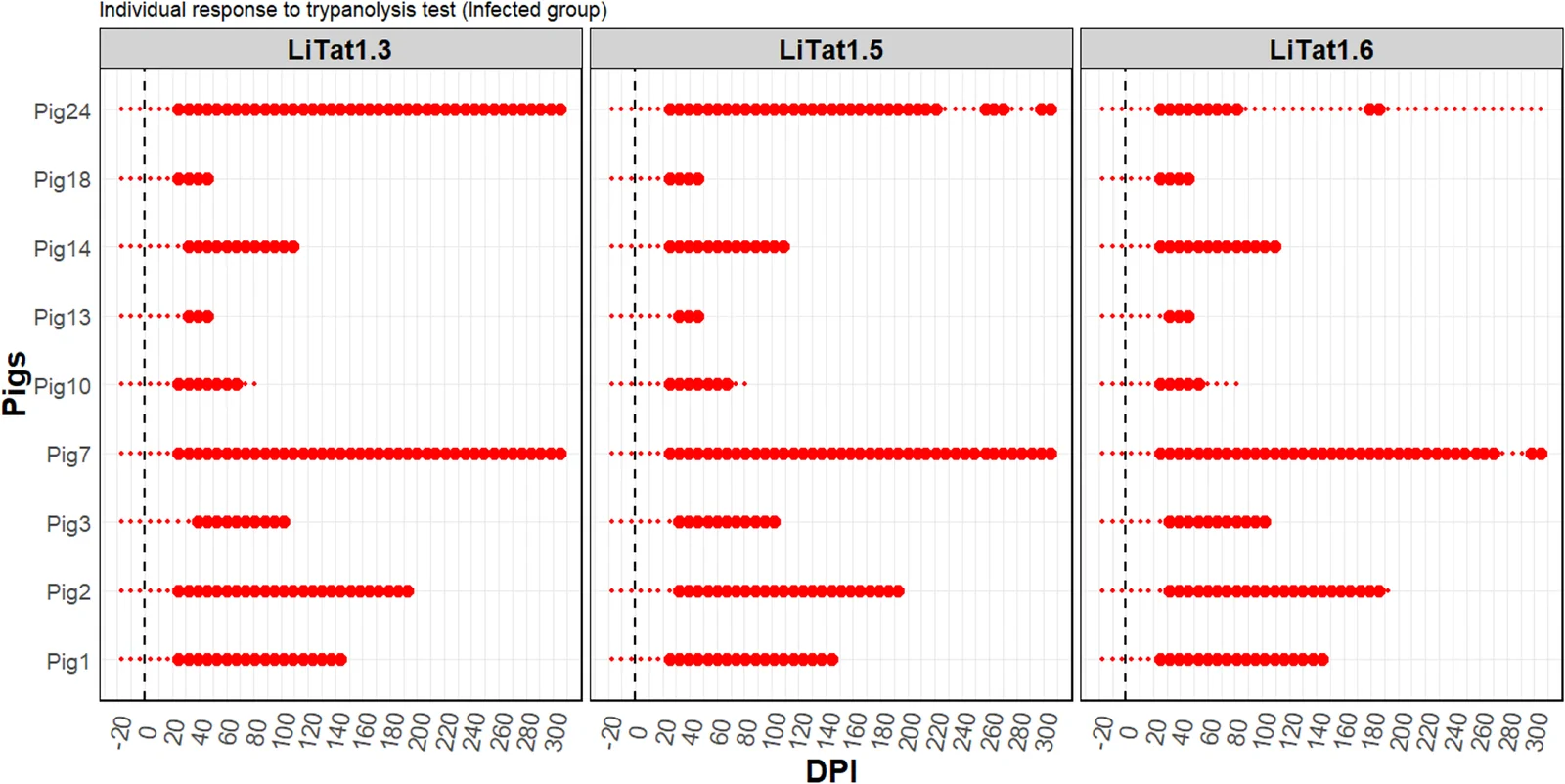
TL results per infected pig for the three VATs. Large red dot: TL positive, small red dot: TL negative, empty: not available (dead animal); DPI: days post-infection.

### Sensitivity, specificity, and agreement of diagnostic tests

Specificity and sensitivity of the diagnostic tests are shown in Table 2. Only three tests showed a specificity lower than 100% (470/470), due to positive results from the control and pre-infection samples: CATT (272/275; 99%), M18S-II (273/275; 99%), and TBR PCR/DNeasy (268/275; 97%). Sensitivity was variable according to the tests, DNA extraction methods, and infection period. The lowest sensitivity was observed with the single round TgsGP with a maximum of 3% (3/105) during late infection and using the DNeasy Kit. All other tests yielded a sensitivity ranging from 5% (2/36) to 53% (19/36) during the early infection period (4–28 DPI). The highest sensitivity for the serological test during this period was obtained with the CATT (11/36; 31%), and for molecular tests, with the PCR TBR/DNeasy (19/36; 53%). These two tests were more sensitive than parasitology by BCT (20/72; 28%). Both the ELISA and AnTat A/B PCR gave a particularly low sensitivity (2/36; 6%).

**Table 2:** Specificity and sensitivity of the diagnostic tests.

Tests	*n* negative / *N* uninfected	Specificity (95% CI)	Early infection (DPI 4–28)		Late infection (DPI 32–147)	
			*n* positive / *N* infected	Sensitivity (95% CI)	*n* positive / *N* infected	Sensitivity (95% CI)
BCT	470/470	100% (99–100)	20/72	28% (18-40)	71/207	34% (28–41)
TBR PCR (Chelex)	275/275	100% (99–100)	11/36	31% (16–48)	42/105	40% (31–50)
TBR PCR (DNeasy)	268/275	97% (95–99)	19/36	53% (36–70)	95/105	90% (83–95)
Single round TgsGP PCR (Chelex)	275/275	100% (99–100)	0/36	0% (0–9)	0/105	0% (0–4)
Single round TgsGP PCR (DNeasy)	275/275	100% (99–100)	0/36	0% (0–9)	3/105	3% (1–9)
Nested TgsGP PCR	275/275	100% (99–100)	9/36	25% (12–42)	50/105	48% (38–58)
LiTat A/B PCR	275/275	100% (99–100)	9/36	25% (12–42)	39/105	37% (28–47)
Antat A/B PCR	275/275	100% (99–100)	2/36	6% (1–2)	13/105	12% (7–20)
M18S-II PCR	273/275	99% (97–100)	12/36	33% (19–51)	55/105	52% (42–62)
RDT	275/275	100% (99–100)	4/36	11% (3–26)	62/105	59% (49–69)
CATT	272/275	99% (96–100)	11/36	31% (16–48)	100/105	95% (89–98)
ELISA	275/275	100% (99–100)	2/36	6% (1–2)	77/105	73% (64–82)
LiTat1.3 TL	275/275	100% (99–100)	6/36	17% (6–33)	102/105	97% (92–99)
LiTat1.5 TL	275/275	100% (99–100)	6/36	17% (6–33)	103/105	98% (93–100)
LiTat1.6 TL	275/275	100% (99–100)	6/36	17% (6–33)	92/105	87% (80–93)

*n* positive: number of positive samples; *n* negative: number of negative samples; *N* infected: number of samples from infected pigs; *N* uninfected: number of samples from uninfected pigs; CI: confidence interval.

For all tests, sensitivity was higher during the infection (32–147 DPI). However, it remained weak for the BCT (71/207; 34%). Sensitivity was also low for the molecular tests (between 13/105; 12% and 55/105; 52%), except for the TBR PCR/DNeasy (95/105; 90%). All serological tests other than the RDT (59%) showed high sensitivity (> 73%). This sensitivity reached 95% (100/105) to 98% (103/105) for the CATT, the LiTat 1.3, and LiTat 1.5 TL.

## Discussion

Pigs are considered a potential animal reservoir for *T. b. gambiense* and may therefore contribute to the re-emergence of HAT. As a result, it is essential to improve our knowledge of the dynamics of *T. b. gambiense* infection in pigs. Our long-term monitoring study of *T. b. gambiense* experimental infections in pigs showed that the classical parasitology method detects the infection in only one third of specimens. While PCR sensitivity reached up to 90% when targeting the *Trypanozoon*-specific TBR multicopy genes, we confirmed the difficulty to detect the *T. b. gambiense* subspecies with the most commonly used specific primers. Serology, especially CATT and TL with LiTat 1.3 and 1.5, detects trypanosome infection relatively well.

The major limitation of this study was the superinfection of pigs with *colibacillus*, *Coccidia* and *Ascaris*, and potentially other pathogens. These infections resulted in clinical symptoms and signs that prevented us from observing those specifically caused by *T. b. gambiense,* and were probably responsible for the death of most pigs, reducing the number and reliability of the results. In retrospect, stricter quarantine and health screening on arrival (including deworming and antibiotic prophylaxis), closer monitoring of diet, and earlier veterinary intervention at the first clinical signs may help limit such background morbidity in future long-term experimental husbandry of pigs. The other infections probably also had an impact on the performance of the serological diagnostic tests. Despite the superinfections, from a clinical point of view, it seems that the infection with *T. b. gambiense* did not have a significant impact on the weight, PCV, and symptoms of the animals. This confirms previous observations that describe pigs as tolerant of *T. brucei* infections [25, 34, 39]. However, as both infected and control groups experienced substantial background disease, this apparent lack of clinical impact should not be taken as strong evidence of low pathogenicity of *T. b. gambiense* in pigs. A cleaner health status at the outset would be needed to draw firmer conclusions on this point.

The parasitological and molecular methods confirmed the successful establishment of *T. b. gambiense* in pigs as a long-lasting infection. Parasitological testing detected the presence of trypanosomes between 17 and 182 DPI, with a peak in the two-month period post-infection, followed by gradual disappearance of the parasites. We cannot exclude that the use of mAECT on BC [6], which recently showed a higher sensitivity during a pig survey in Côte d'Ivoire [49], would have resulted in more positive observations. Overall, PCR methods confirmed parasitological observations with earlier and later detection of trypanosomes. DNA extraction with the DNeasy Kit seems preferable to purification methods such as Chelex for better analytical sensitivity. The TBR PCR yielded the highest overall positivity rates and detected parasite DNA up to 305 DPI. Unfortunately, it also presented the lowest specificity and did not make it possible to distinguish *T. b. gambiense* from *T. b. brucei*. The Antat PCR showed the lowest sensitivity and did not appear to be suitable for *T. b. gambiense* detection in pigs. Our results confirm the low sensitivity of the TgsGP single round PCR, which can be slightly improved using nested PCR, which nonetheless has limited specificity, as recently described in several studies [19, 49]. In our research, this limited specificity could be due to random contamination by small quantities of *T. brucei* DNA during the extraction and PCR steps. Contamination of this type may occur in all laboratories that routinely work with such DNA, particularly when nested PCR is used to increase sensitivity, even when all experiments are validated by negative controls. Therefore, these results not only confirm that the pig is sensitive to *T. b. gambiense* infection, but indicate that the pig can remain infected, and thus act as a reservoir of this parasite for at least one year. Our results are inconsistent with a previous experimental study that observed a spontaneous cure of domestic pigs experimentally infected in fewer than 6 months [41]. In this study, pigs were susceptible to *T. b. gambiense* with a shorter prepatent period, which was probably due to immunosuppression before infection. After two months, parasites gradually disappeared and the TBR PCR became negative almost at the same time as parasitology test results, between 83 and 115 DPI. This could be due to the DNA purification method used in this study (Ready AMP™ genomic purification kit, Promega, Madison, WI, USA) that may have limited the sensitivity of the PCR. Here, we show that the PCRs performed on DNA purified with Chelex were less sensitive than those performed on the DNA extracted with the DNeasy Kit. Unfortunately, we cannot rule out the fact that this difference could be due, at least in part, to the other infections observed during our study. Nevertheless, our results now clearly show that *T. b. gambiense* infections in pig can persist for a long time, which supports the candidacy of this animal as a long-term reservoir.

Low parasitaemia, as observed in the present study, is a known characteristic of *T. b. gambiense* infections [34]. A comparison with our previous experimental *T. b. brucei* infection study in pigs [20, 21] further suggests subspecies-specific infection dynamics. Of note, *T. b. brucei* infection was characterised by a shorter prepatent period (BCT-positive from 10–14 DPI on average, versus 17–32 DPI in the present study), high average and maximum parasitaemia (10^4^ and 2.5 × 10^6^ tryp/mL, respectively), and trypanosomes detected over a long period [21]. In contrast, *T. b. gambiense* infection resulted in lower parasitaemia and a gradual disappearance of detectable parasites from the blood, despite a persistent infection as witnessed by PCR. Further comparative studies are required to improve our knowledge of the dynamics of *T. b. gambiense* infection in pigs, taking into account that trypanosomes can colonise tissues other than blood, such as the dermis and adipose tissue, as recently observed in *T. b. brucei* experimental infection in mice [8, 50, 51] or during HAT field studies [7, 27, 45].

Serological tests became positive in the weeks following the infection. The CATT proved to be a sensitive method for indirect detection of early and late infection, with good diagnostic specificity. The TL using the LiTat 1.3 and 1.5 VAT showed high sensitivities in late infection. In the present *T. b. gambiense* infection, we observed a rapid and simultaneous response against the three VATs, in contrast to the earlier *T. b. brucei* experimental infections in pigs, showing a sequential antibody response, first against LiTat 1.6, then LiTat 1.5, and lastly LiTat 1.3 [21]. As expected, the CATT, RDT, and TL remained mostly positive until the death of the pigs, since antibodies can persist for some time after infection, even after treatment or self-cure [13]. The ELISA method, previously developed for the detection of *T. b. brucei* [20], showed acceptable sensitivity, with some pigs that were good responders, and others not. Four pigs suffering from the presence of other pathogens and early mortality, did not develop a trypanosome-specific antibody response detectable by the indirect ELISA method, unlike *T. b. gambiense* infected pigs that survived longer and remained positive until the end of the infection. Since ELISA detects IgG, it can be hypothesised that these 4 pigs probably failed to develop an effective trypanosome-specific IgG response. The other serological tests (CATT, RDT, and TL) are probably rather IgM-dependent. Under optimal experimental conditions without risk of co-infection, indirect ELISA seems appropriate for long-term experimental follow-ups.

Several limitations of the present study should be acknowledged. Infections were established using a single *T. b. gambiense* isolate (B4F303). Infection dynamics, including virulence, pathogenicity and diagnostic test performance may vary among strains, and our findings should be generalised with caution. The high attrition of pigs due to intercurrent infections also left only a small number of animals under long-term follow-up, so conclusions on very long-term carriage remain only for a limited number of surviving pigs. Finally, infection was established by intradermal inoculation of a mouse-amplified strain, which may not fully reproduce the dose, kinetics, and tissue tropism of natural tsetse-transmitted infection. This should be considered when extrapolating our findings to field conditions.

Overall, our experimental results suggest that pigs can remain infected with *T. b. gambiense* for an extended period. They confirm that this species should be considered a potential reservoir of the parasite. Whether such long-term carriage translates into genuine epidemiological importance, however, remains an open question. Future studies incorporating xenodiagnosis or the recovery of viable parasites from tissues would provide stronger evidence of transmissibility and reservoir competence. Although a reservoir of this kind has not prevented the elimination of the disease as a public health problem in several HAT foci in recent years, it could jeopardise maintenance of this achievement [23]. The present study focused on pigs as a candidate domestic reservoir, given their frequent multi-infection with *T. brucei* and their proximity to human dwellings in HAT foci [15, 29, 39, 49]. This does not exclude a role for wild animal reservoirs, which remain comparatively understudied. This question was beyond the scope of the present experimental study but should be addressed in future field surveys [4]. As an additional example, stakeholders involved in Guinea worm control now regret not having considered dogs as a potential reservoir earlier, as canine infections now threaten progress towards eradication [43]. Improving our knowledge through experimental studies and field surveys will allow for the development and selection of more efficient diagnostic tools, notably to differentiate between *T. b. brucei* and *T. b. gambiense*. To improve our knowledge of the dynamics of infection of the two subspecies, it appears essential to test for trypanosomes in the whole body, i.e. in all tissues in which they might survive, and in particular, dermal tissues and deep organs [1, 7, 45]. The presence of trypanosomes in host tissues may increase the risk of long-term carriage of the parasites, with a possible impact on their transmission to another host. The use of xenomonitoring, using naive tsetse flies as previously described [48], would be informative to consider this risk of transmissibility. It would also be valuable to conduct new experimental infections considering co-infection with *T. b. brucei* and *T. b. gambiense* to determine how they can interact, as this could occur in HAT foci. It would also be interesting to identify *T. brucei*-infected pigs in HAT foci and to transfer them to a research centre for experimental follow-up using the same diagnostic methods, assessing long-term carriage and diffusion in tissues.

While further evidence is gathered, various measures could be incorporated into control strategies in order to progress towards sustainable elimination of HAT. One solution would be to limit contact between pigs and tsetse flies, by implementing vector control and restricting the method of free-range rearing. This requires a socio-anthropological approach to convince farmers, considering that they do not necessarily perceive the risk posed by their pigs, which themselves tolerate the disease very well. The treatment of pigs could also be considered, but it would require the identification of a treatment regimen (dosage and frequency) that is effective against *T. brucei* sp. and adapted to this animal, noting that trypanocides such as diminazene aceturate often fail to fully clear infection, including from extravascular niches and the central nervous system.

## Figures and Tables

**Supplementary Figure S1:**
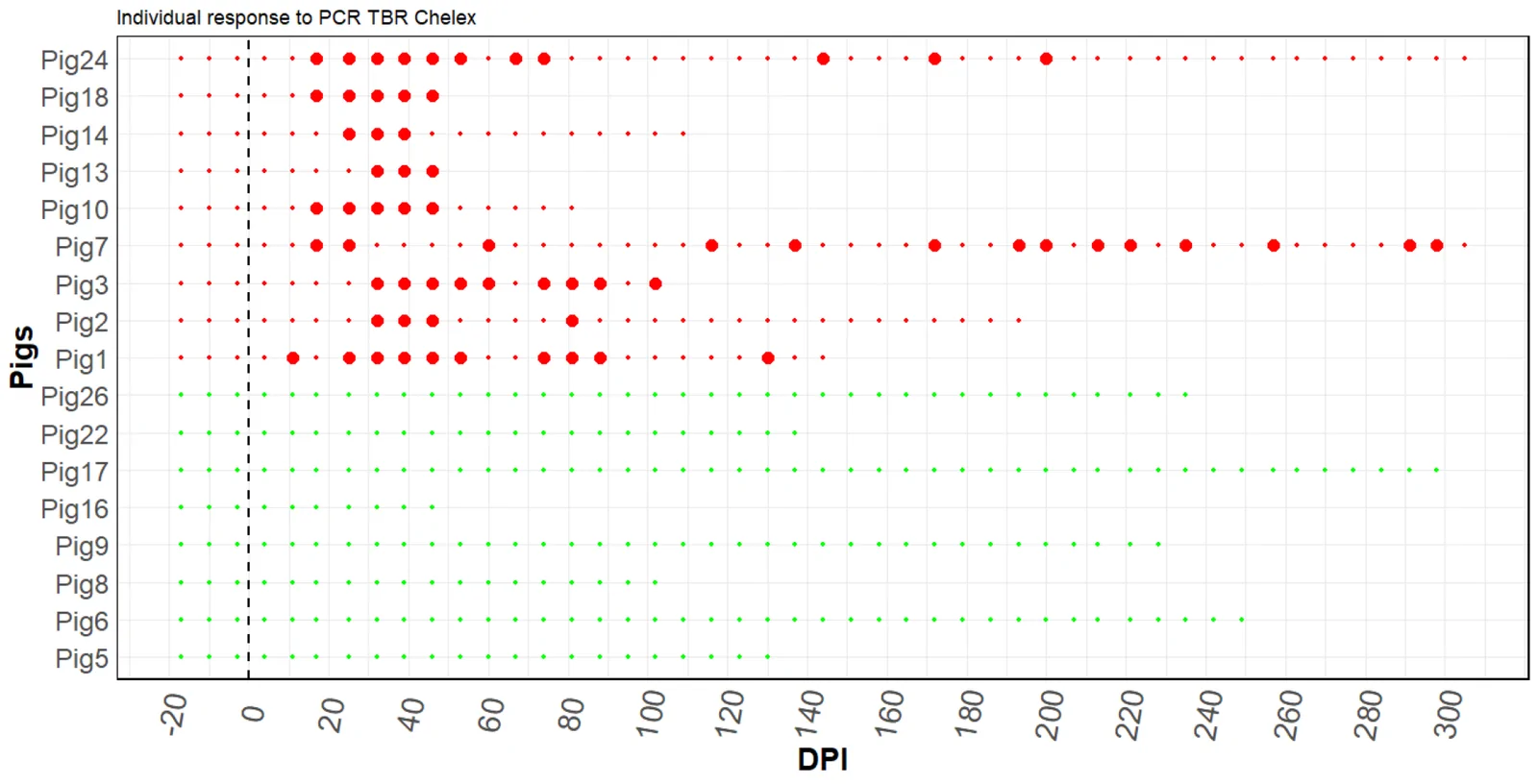
TBR PCR results according to the Chelex DNA extraction method for individual pigs. Large red and green dots indicate positive tests; small red and green dots indicate negative tests. DPI Days post-infection. All red dots indicate infected group and green dots control group.

**Supplementary Figure S2:**
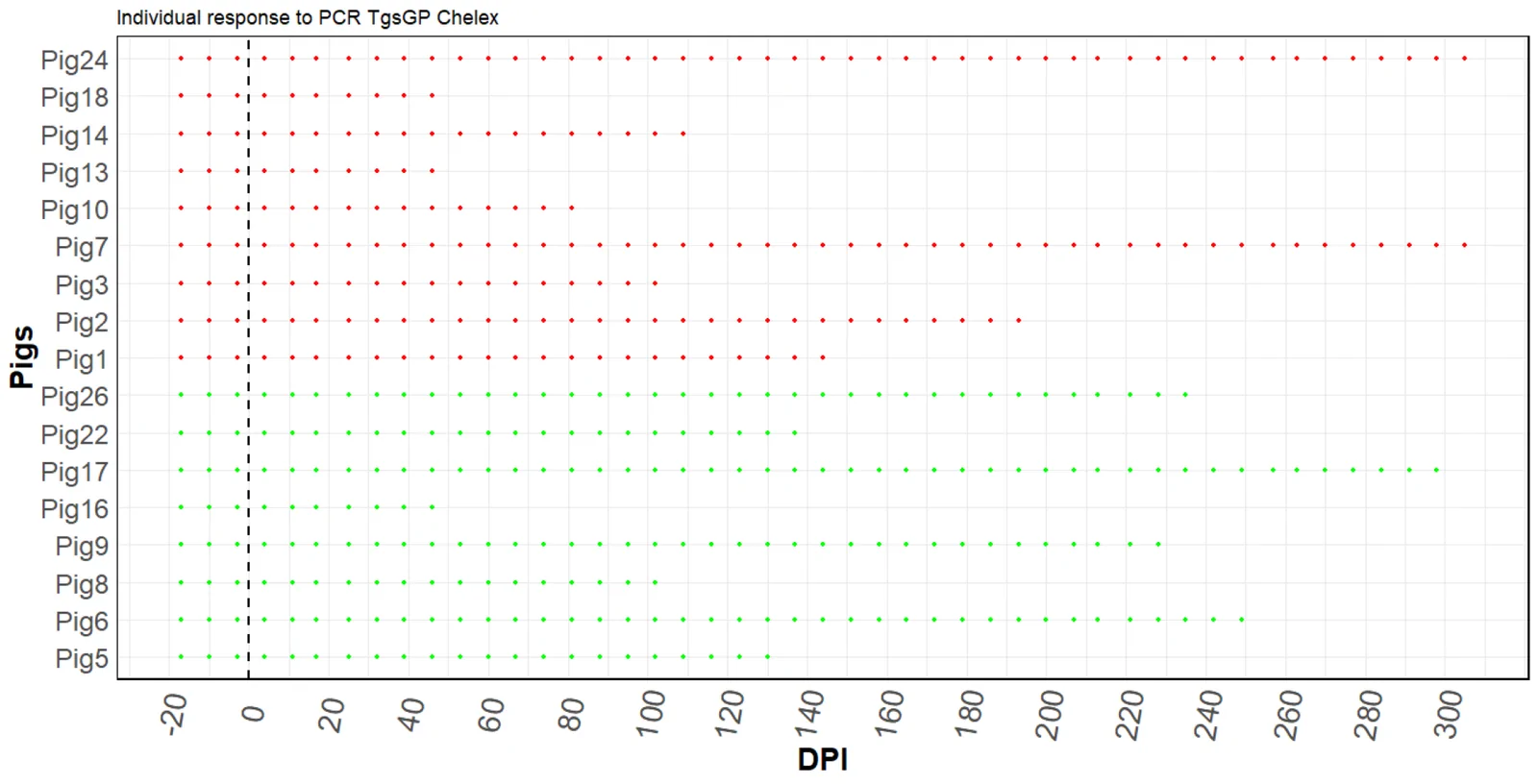
TgsGP PCR results according to the Chelex DNA extraction method for individual pigs. Large red and green dots indicate positive tests; small red and green dots indicate negative tests. DPI Days post-infection. All red dots indicate infected group and green dots control group.

**Supplementary Figure S3:**
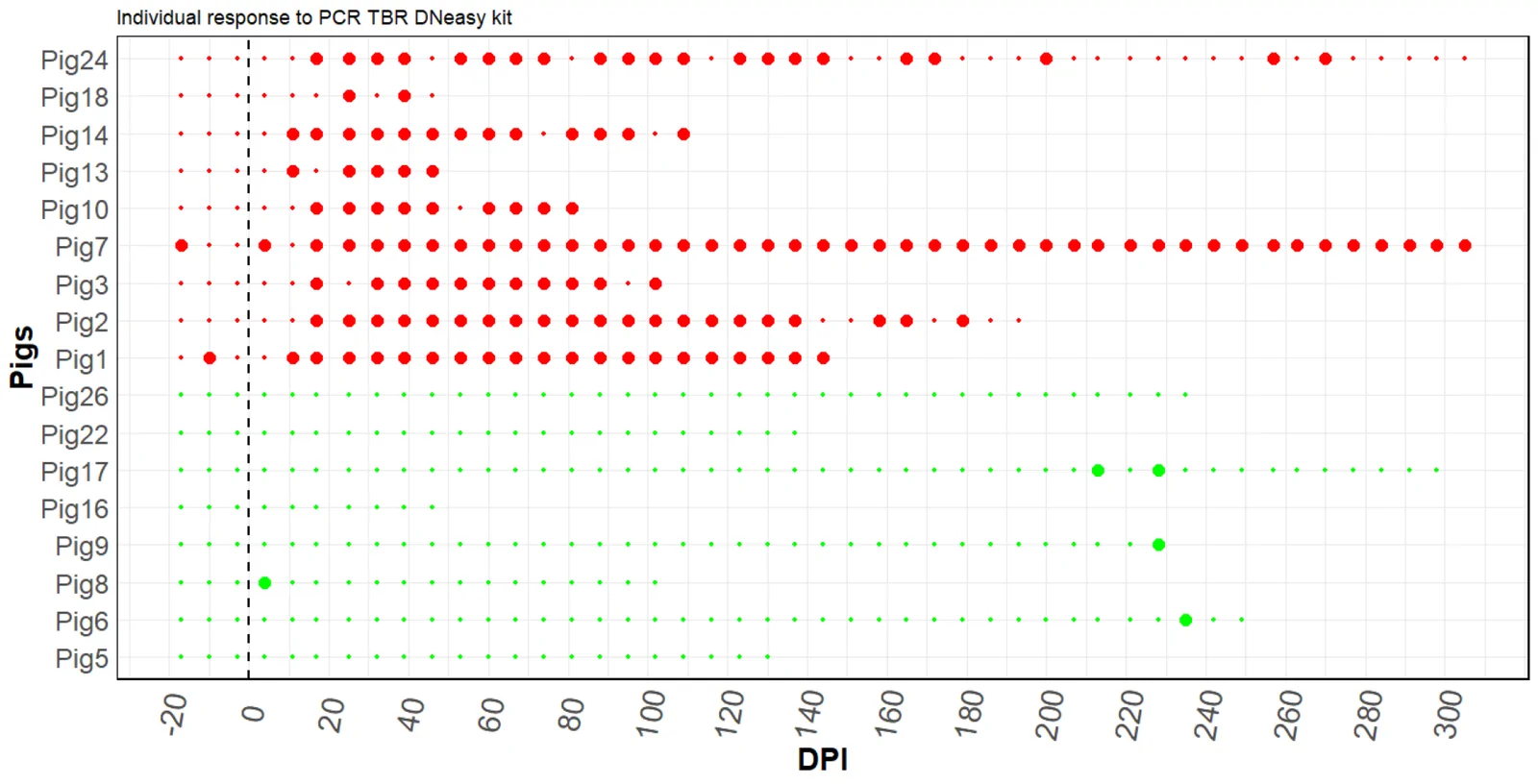
TBR PCR results according to the DNeasy Kit DNA extraction method for individual pigs. Large red and green dots indicate positive tests; small red and green dots indicate negative tests. DPI Days post-infection. All red dots indicate infected group and green dots control group.

**Supplementary Figure S4:**
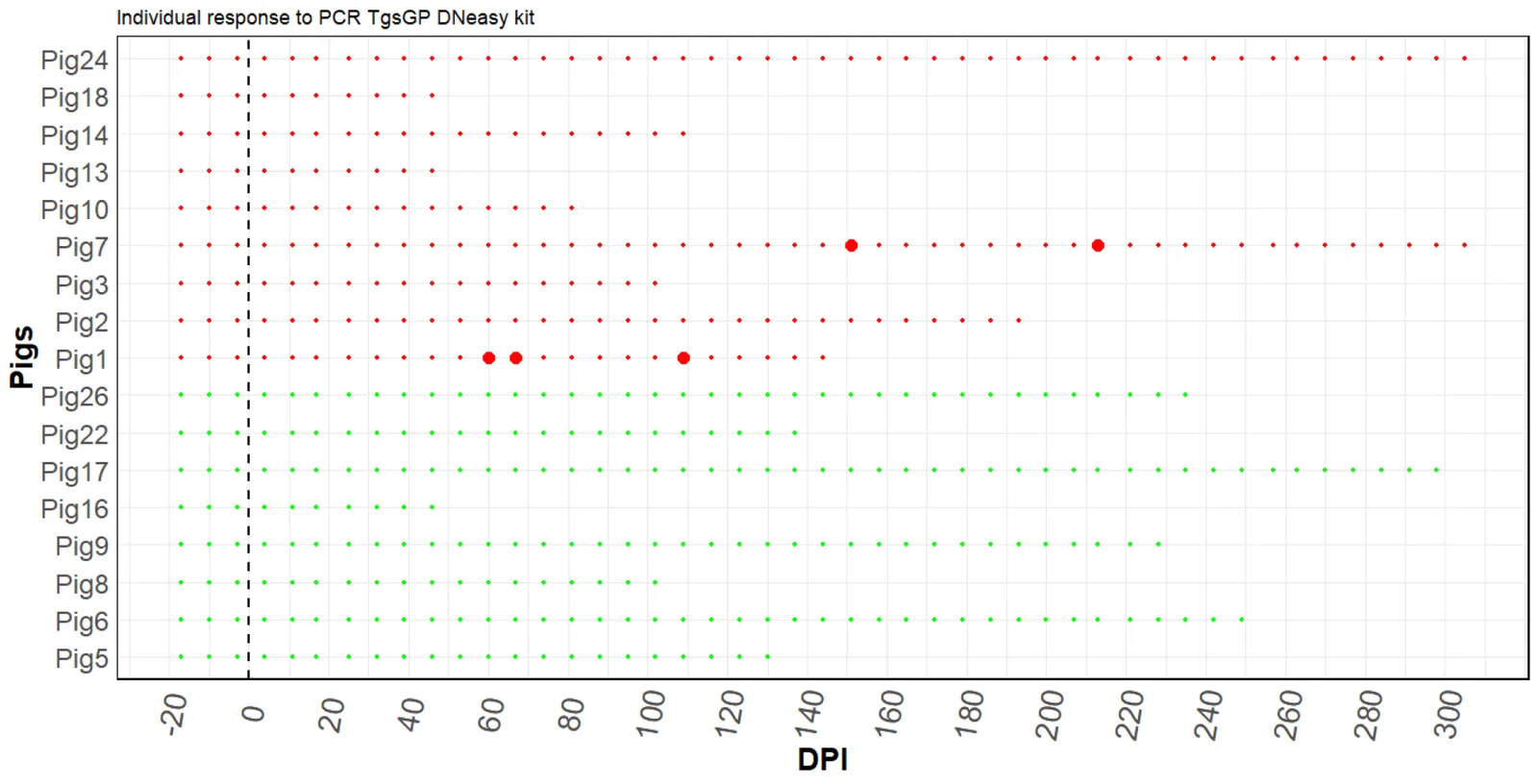
TgsGP PCR results according to the DNeasy Kit DNA extraction method for individual pigs. Large red and green dots indicate positive tests; small red and green dots indicate negative tests. DPI Days post-infection. All red dots indicate infected group and green dots control group.

**Supplementary Figure S5:**
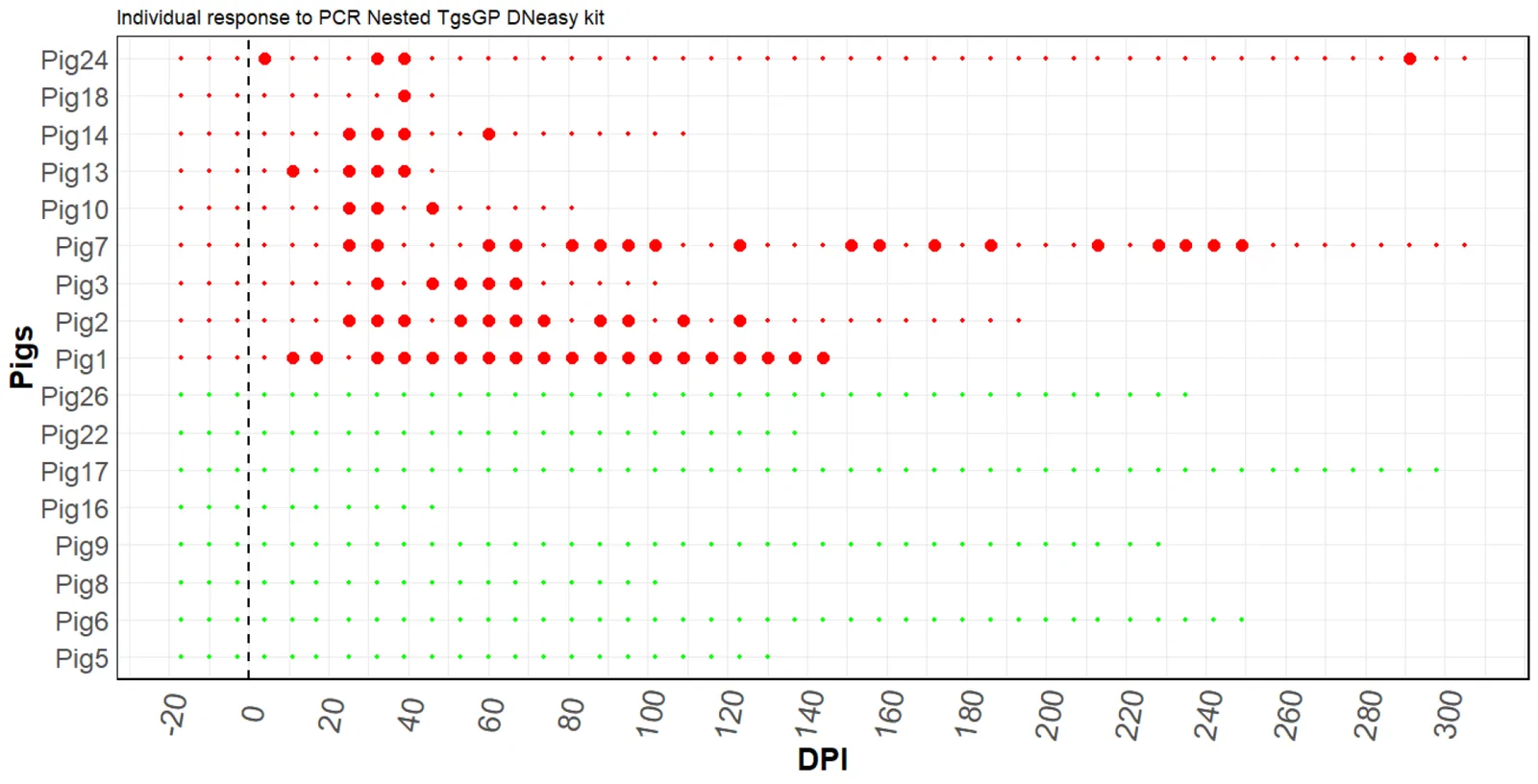
Nested TgsGP PCR results according to the DNeasy Kit DNA extraction method for individual pigs. Large red and green dots indicate positive tests; small red and green dots indicate negative tests. DPI Days post-infection. All red dots indicate infected group and green dots control group.

**Supplementary Figure S6:**
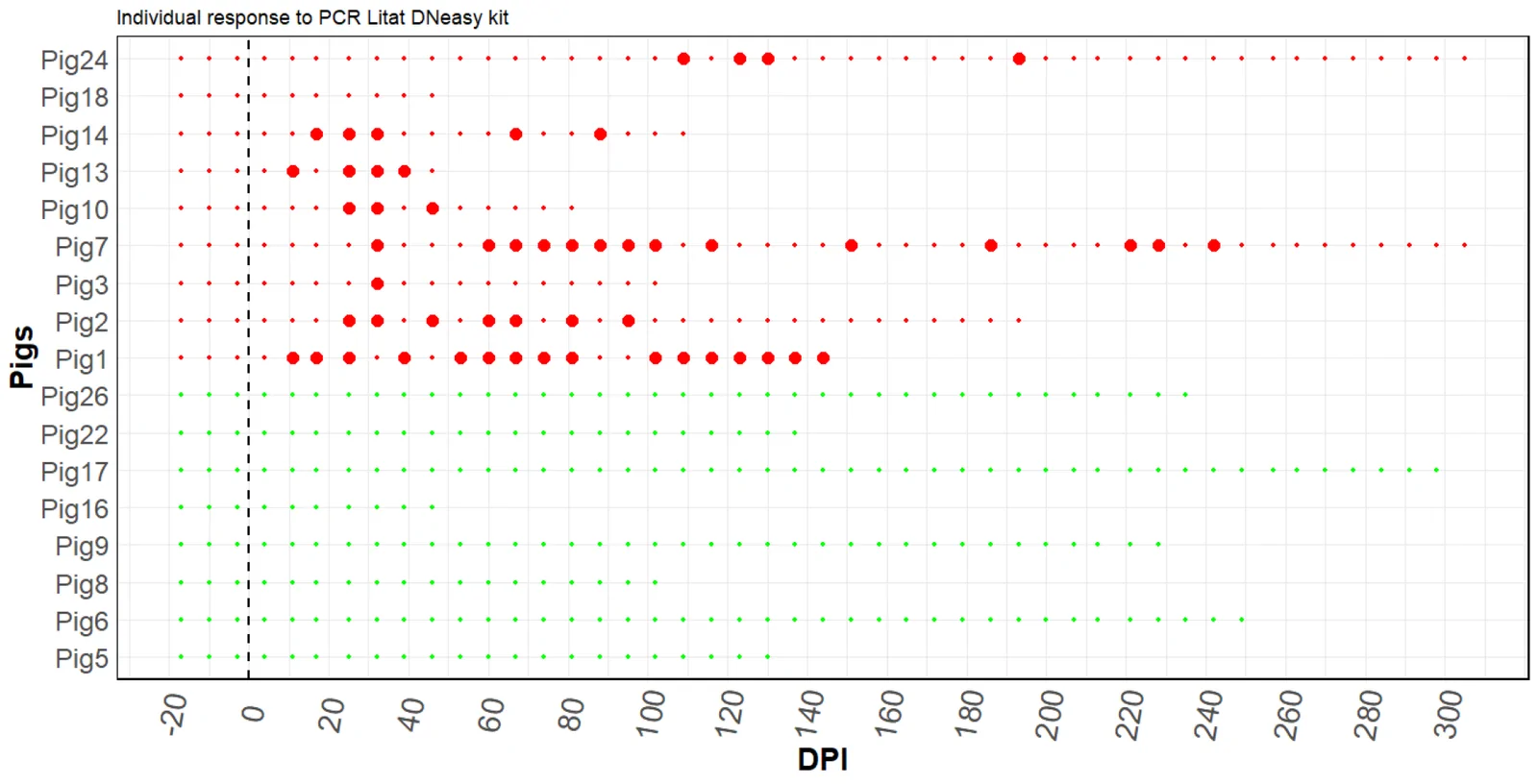
LiTat A/B PCR results according to the DNeasy Kit DNA extraction method for individual pigs. Large red and green dots indicate positive tests; small red and green dots indicate negative tests. DPI Days post-infection. All red dots indicate infected group and green dots control group.

**Supplementary Figure S7:**
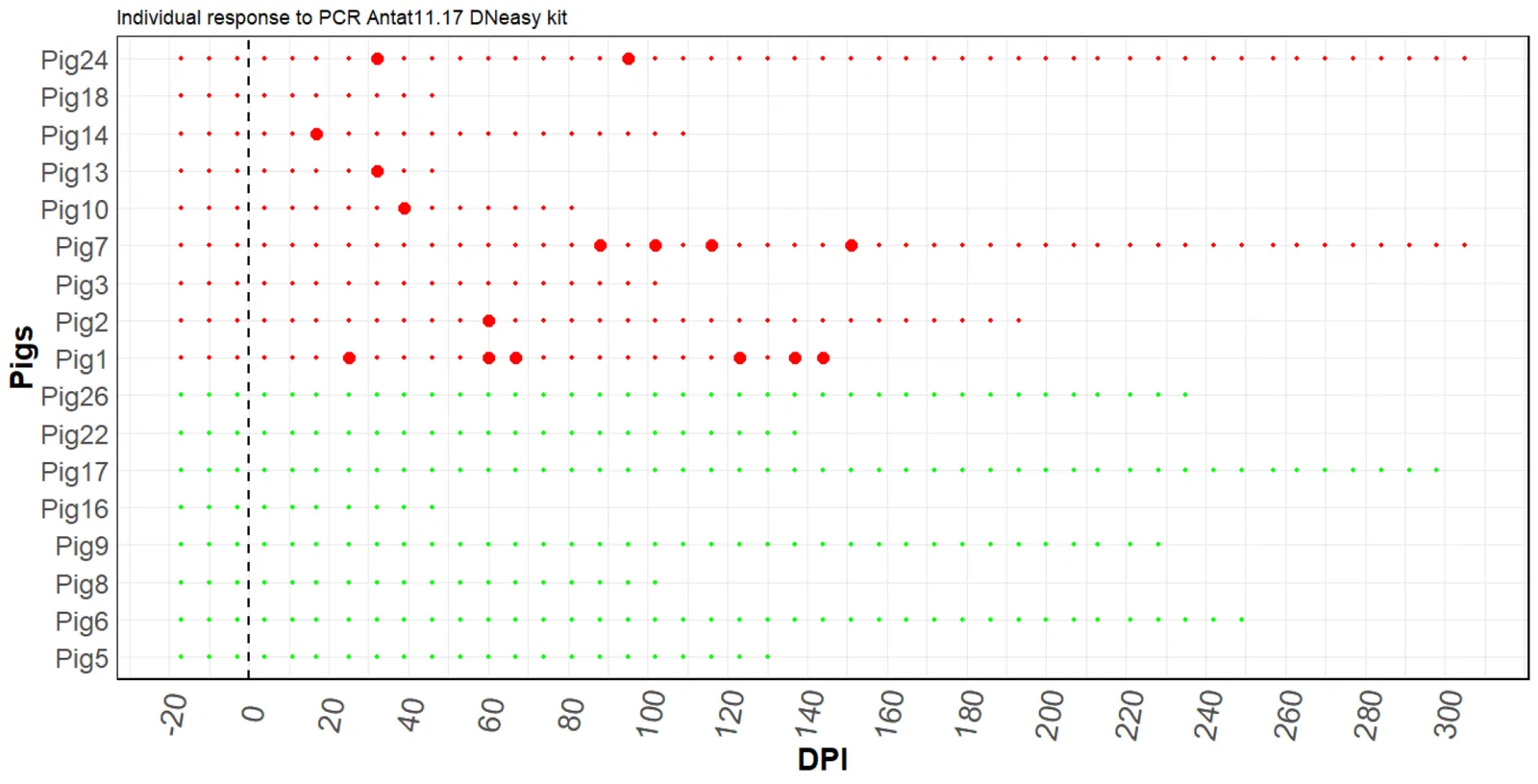
AnTat A/B PCR results according to the DNeasy kit DNA extraction method for individual pigs. Large red and green dots indicate positive tests; small red and green dots indicate negative tests. DPI Days post-infection. All red dots indicate infected group and green dots control group.

**Supplementary Figure S8:**
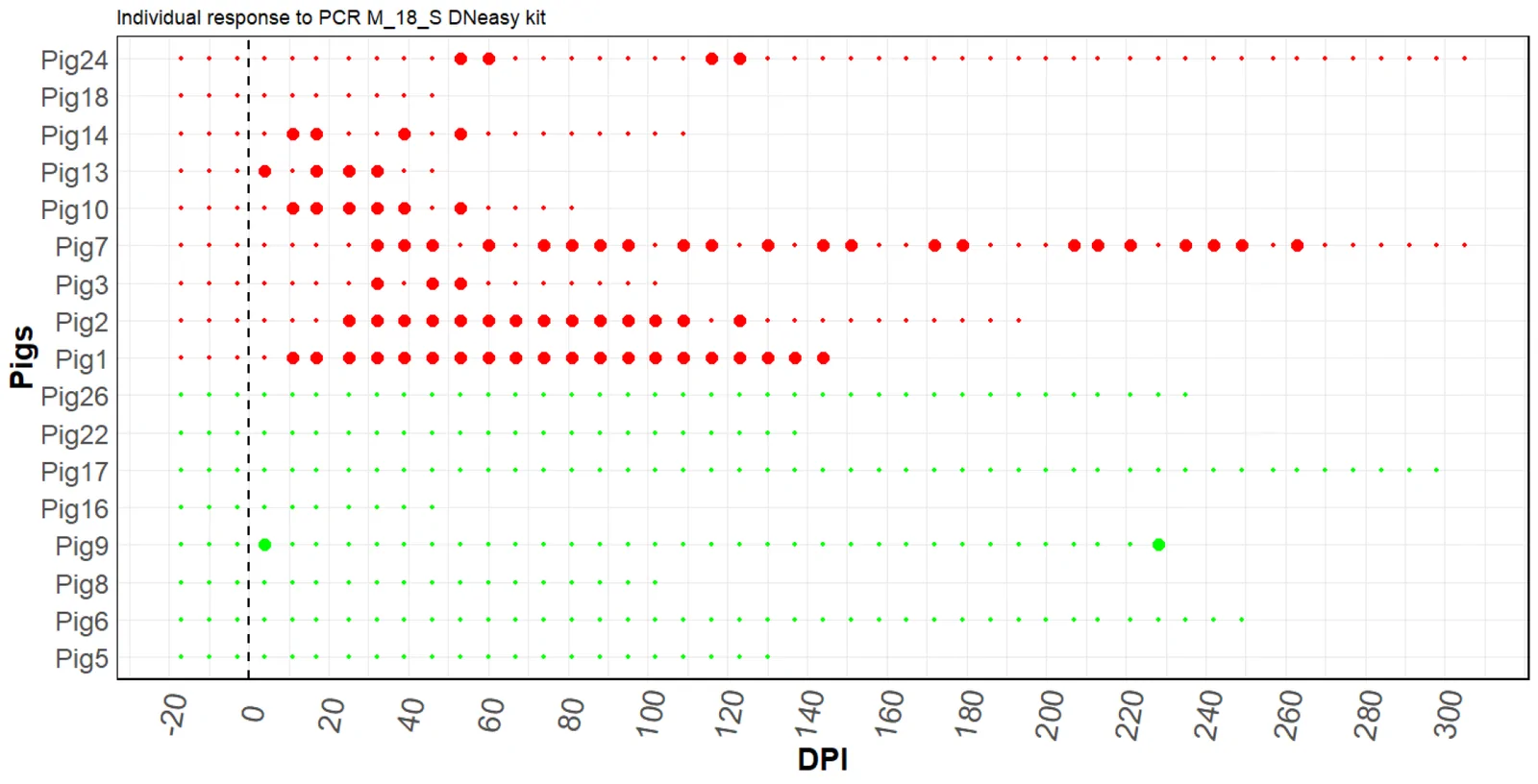
M18S-II PCR results according to the DNeasy Kit DNA extraction method for individual pigs. Large red and green dots indicate positive tests; small red and green dots indicate negative tests. DPI Days post-infection. All red dots indicate infected group and green dots control group.

## Supplementary materials

*Supplementary Data 1*: Raw data for clinical signs. Date: date of observation; DPI: Days post-infection; Group: group C for the control group, I for infected group; Pig: pig identifier; Clinical signs: Symptoms and signs observed; Phase: pre_inf for before infection; post_inf for after infection. *Supplementary Data 2*: Raw data for animal survival. Date: date of death; DPI: Days post-infection; Group: group C for the control group, I for infected group; Pig: pig identifier. *Supplementary Data 3*: Raw data for packed cell volume (PCV). Date: date of sampling; DPI: Days post-infection; Group: group C for the control group, I for infected group; Pig: pig identifier; PCV: animal packed cell volume (PCV); Phase: pre_inf for before infection; post_inf for after infection. *Supplementary Data 4*: Raw data for weight. Date: date of sampling; DPI: Days post-infection; Group: group C for the control group, I for infected group; Pig: pig identifier; Weight: animal weight; Phase: pre_inf for before infection; post_inf for after infection. *Supplementary Data 5*: Raw data for the buffy coat test. Date: date of sampling; DPI: Days post-infection; Group: group C for the control group, I for infected group; Pig: pig identifier; BCT_Score: parasitaemia score; Tryp.mL: Number of trypanosomes/mL; Log10_ Tryp.mL: log10 of parasitaemia in trypanosomes/mL; Result: result of the BCT test 0 for negative, 1 for positive; Phase: pre_inf for before infection; post_inf for after infection. *Supplementary Data 6*: Raw data for PCR tests. Date: date of sampling; DPI: Days post-infection; Group: group C for the control group, I for infected group; Pig: pig identifier; TBR_Chelex, TgsGP_Chelex, TBR_DNeasy, TgsGP_DNeasy, NestedTgsGP_DNeasy, AntatA.B_DNeasy, LitatA.B_DNeasy and M18_S_DNeasy: result of the TBR, single round TgsGP, Nested TgsGP, AntatA/B, LitatA/B and M18_S PCR. 0 for negative, 1 for positive; Phase: pre_inf for before infection; post_inf for after infection. *Supplementary Data 7*: Raw data for serological tests. Date: date of sampling; DPI: Days post-infection; Group: group C for the control group, I for infected group; Pig: pig identifier; Result_CATT, Result_TDR, Result_LiTat1.3; Result_LiTat1.5, Result_LiTat1.6 and Result_ELISA: result of the serological (CATT, RDT, ELISA, TL LiTat1.6) test 0 for negative, 1 for positive; OD_moy_ELISA: Optical Density; Phase: pre_inf for before infection; post_inf for after infection. *Supplementary Data 8*: Script with details of the data analysis.

The supplementary material of this article is available at https://www.parasite-journal.org/10.1051/parasite/2026061/olm.
